# Uncoupling protein 2 mediates resistance to gemcitabine-induced apoptosis in hepatocellular carcinoma cell lines

**DOI:** 10.1042/BSR20150116

**Published:** 2015-07-29

**Authors:** Guangsheng Yu, Jun Liu, Kesen Xu, Jiahong Dong

**Affiliations:** * Department of Hepatobiliary Surgery, Shandong Provincial Hospital affiliated to Shandong University, Jinan 250021, China; † Department of Hepatobiliary Surgery, Qilu Hospital of Shandong University, Jinan 250012, China; ‡ Department of Hepatobiliary Surgery, Chinese General PLA Hospital, Beijing 100853, China

**Keywords:** chemoresistance, hepatocellular carcinoma (HCC), uncoupling protein 2 (UCP2), gemcitabine

## Abstract

Oxidative stress induction is a common effector pathway for commonly used chemotherapeutic agents like gemcitabine (GEM) in hepatocellular carcinoma (HCC) patients. However, GEM alone or in combination with oxiplatin hardly renders any survival benefits to HCC patients. Mitochondrial uncoupling protein 2 (UCP2) is known to suppress mitochondrial reactive oxygen species (ROS) generation, thus mitigating oxidative stress-induced apoptosis. We demonstrate in the present study, using a panel of HCC cell lines that sensitivity to GEM in HCC well correlate with the endogenous level of UCP2 protein expression. Moreover, ectopic overexpression of *UCP2* in a HCC cell line with low endogenous UCP2 expression, HLE, significantly decreased mitochondrial superoxide induction by the anti-cancer drug GEM. Conversely, *UCP2* mRNA silencing by RNA interference in HCC cell lines with high endogenous UCP2 expression significantly enhanced GEM-induced mitochondrial superoxide generation and apoptosis. Cumulatively, our results suggest a critical role for mitochondrial uncoupling in GEM resistance in HCC cell lines. Hence, synergistic targeting of *UCP2* in combination with other chemotherapeutic agents might be more potent in HCC patients.

## Abstract (precis)

Our results suggest a critical role for mitochondrial uncoupling in gemcitabine (GEM) resistance in hepatocellular carcinoma (HCC) cell lines. Hence, synergistic targeting of uncoupling protein 2 (*UCP2*) in combination with other chemotherapeutic agents might be more potent in HCC patients.

## INTRODUCTION

The uncoupling protein (UCP) family is a sub-category of mitochondrial anion-carrier proteins super-family and can be found in both animal and plant species [1]. Genome of mammalian species encode five UCP homologues (UCP1–5), with UCP1–3 demonstrating high sequence similarity [1–3]. UCPs localize to the inner membrane of mitochondria and were originally considered to regulate thermogenic proton leak [1]. Compared with UCP1 and UCP3 that are expressed more in the brown adipose tissue and skeletal muscle [1,4,5], expression of UCP2 is more ubiquitous and can be detected in liver, brain, pancreas, central nervous system and immune cells [4].

Whereas UCP1 is a key regulator of adaptive thermogenesis, UCP2 and UCP3 also process important function to decrease the reactive oxygen species (ROS) that is produced by electron transport [6,7]. ROS is natural byproduct of the normal oxygen metabolism. In physiological condition, it had been demonstrated that ROS is involved in cell signalling processes, such as cell proliferation, inflammation, apoptosis and phagocytosis [8]. However, ROS level can be dramatically up-regulated during the stress condition to cause oxidative stress which result in oxidative damage to the cell [9]. Therefore, since UCPs function to modulate ROS, which is involved in regulation of cell survival, it can be hypothesized that UCPs may be involved in the progression of cancer [10]. Indeed, UCP2 expression is up-regulated in several hepatocellular carcinoma (HCC) cell lines [11], as well as human colon cancer [12]. It had been demonstrated that ROS levels is also correlated with up-regulated UCP2 and UCP5 expression in colon cancer cells [13], which implies that elevated UCPs may be due to increased oxidative stress. Moreover, increasing evidences also suggest that p53 is a functional target of UCP2 [14]. The cellular oxidative stress level might lead p53 to downstream pro-apoptotic pathways [15]. The modulation of ROS by UCP2 is likely to alter p53 responses and assist the survival of cancer cells [14]. Taken together, these data suggests UCP2 may be a novel therapeutic target in different solid cancers. However, the precise role UCP2 in HCC is still largely unknown.

Gemcitabine (2′,2′-difluoro-2′-deoxycytidine; GEM) is used in combination with oxiplatin as chemotherapeutic agents in HCC. However, using GEM rarely results in survival benefits to a patient with HCC, highlighting the need for identification of novel targets that dictate response to chemotherapy, the regulation of which in turn may improve sensitivity to GEM. Two evidences suggest that UCP2 targeting might be a potential therapeutic strategy for HCC, (a) *UCP2* overexpressing colon cancer cells are more resistant to CPT-11, ((4S)-4,11-diethyl-3,4,12,14-tetrahydro-4-hydroxy-3,14-dioxo-1H-pyrano[3′,4′:6,7]indolizino[1,2-b]quinolin-9-yl [1,4′-bipiperidine]-1′-carboxylic acid ester hydrochloride), a topoisomerase I inhibitor and (b) genipin, a specific UCP2 inhibitor, enhanced sensitization to anthracyclin to drug-resistant leukaemia cells [16,17]. In the present study, using cellular models of HCC cell lines clinically treated with GEM, we investigated the involvement of *UCP2* expression in rendering cellular resistance to GEM and the anti-tumoral effect of GEM treatment associated to *UCP2* inhibition.

## MATERIALS AND METHODS

### Cell, plasmids, siRNA, transfection and chemicals

HuH6, Hep3B, HepG2 cell and HLE HCC cell lines were purchased from A.T.C.C. and maintained in Dulbecco's Modified Eagle Medium (DMEM) supplemented with 10% FBS (Gibco). Transfection of cell with plasmid DNA or siRNA was performed by using Lipofectamine 2000 (LifeTechnologies), according to the instructions of the manufacturer.

The siRNA targeting *UCP2* was obtained from LifeTechnologies. A scramble siRNA sequence was included as a control. *UCP2* ORF was cloned from cDNA of HepG2 by PCR and ligated to pCDNA3.1 expression vector (LifeTechnologies).

GEM and FCCP (4-trifluoro-methoxy-phenyl-hydrazone) were obtained from Sigma and solubilized in sterile water and 95% ethanol respectively and stored at −20°C until use.

### Isolation of mitochondria

Isolation of mitochondria from different cell lines were as previously described [18]. Briefly, the cells were placed in ice-cold 0.25 mol/l sucrose, 2 mmol/l EDTA, 10 mmol/l Tris/HCl, 5×10^4^ unit/l heparin (SETH) medium and then homogenized. The resulting homogenate was cleared by centrifugation for 10 min at 600 ***g***. The supernatant containing mitochondria was centrifuged again for another 10 min at 14000 ***g*** to pellet the mitochondria. After centrifugation, the mitochondrial pellet was re-suspended in SETH-medium for further analysis.

### Western blot analysis

Cells or mitochondrial extract were lysed by the Laemmli sample buffer as previously described [19,20]. The proteins were resolved by SDS/PAGE and detected by Western blot as previously described [20]. Briefly, after SDS/PAGE, the separated proteins were transferred on to PVDF membrane and probed with rabbit anti-UCP2 antibody (Santa Cruz Biotechnology). Specific reactions were detected using goat anti-rabbit IgG conjugated with horseradish peroxidase (Sigma) and revealed by a chemiluminescence substrate. The membrane was also blotted with GAPDH (glyceraldehyde-3-phosphate dehydrogenase) antibody (Santa Cruz) to confirm equal protein loading. The chemiluminescence signal was recorded by the ChemiDoc XRS imaging system (Bio-Rad Laboratories). The luminescence signal was captured and analysed by using the Quantity One Program (Version 4.6).

### Cell proliferation assays

Cell proliferation was quantified using a mitochondrial colorimetric assay (MTT assay, Sigma–Aldrich) as per the manufacturer's recommendations. The absorbance was measured at 570 nm and post-measurement corrected were performed by subtracting absorbance at the reference wavelength of 690 nm. The results, expressed as relative absorbance (*A*), were obtained for three different experiments and expressed as mean ± S.D.

### Measurement of mitochondrial superoxide production

The non-fluorescent MitoSox Red probe (Molecular Probes, LifeTechnologies) was used to evaluate mitochondrial superoxide production as per manufacturer recommendations. Briefly, 5×10^3^ cells/well were seeded in 96-well plates before being treated with the various compounds at the indicated concentration for 16 h. At the end of the treatments, cells were incubated in culture medium with 0.5 μM MitoSox probe at 37°C for 15 min before being washed with Hanks buffer (20 mM HEPES, pH 7.2, 10 mM glucose, 118 mM NaCl, 4.6 mM KCl and 1 mM CaCl_2_). Fluorescence was measured at excitation of 430 nm and emission of 590 nm. The probe is live-cell permeant and is rapidly and selectively targeted to the mitochondria where it becomes fluorescent after oxidation by O2^•−^. The values were normalized on cell proliferation by MTT.

### Caspase activity assay

Cells/well (5×10^3^) were seeded in 96-well plates and were treated with the various compounds at the indicated concentrations for 48 h. Cells were then labelled in culture with the fluorescent inhibitor/substrate of fluorescent labeled inhibitor of caspases (FLICA) (FAM-DEVD-FMK, Carboxyfluorescein-Asp(OMe)-Glu(OMe)-Val-Asp(OMe)-fluoromethylketone) (Molecular Probes, LifeTechnologies) for 60 min at 37°C. Cells were washed twice with wash buffer and fluorescence measured at excitation of 485 nm and emission of 535 nm. The values were normalized to cell proliferation assessed by MTT.

## RESULTS

### Differential steady state protein expression of UCP2 dictates sensitivity to gemcitabine

To examine the roles UCP2 played in HCC cells, we first checked the endogenous UCP2 expression in a panel of four different HCC cell lines, HuH6, Hep3B, HepG2 and HLE. Whereas, HuH6, Hep3B and HepG2 showed robust endogenous levels of UCP2 protein expression, HLE showed marked suppression of UCP2 expression compared with the other three cell lines (Figure 1A). Interestingly, endogenous UCP2 expression mirrored resistance to GEM-induced cell growth inhibition (Figure 1B) and inhibition of mitochondrial superoxide induction by GEM (Figure 1C). Thus, the HLE cells were the most sensitive to GEM-induced cell growth inhibition and produced the maximum amount of mitochondrial superoxide anion (Figures 1B and 1C). In each case, treatment with GEM in the presence of FCCP, a known chemical uncoupler, significantly induced resistance to GEM-induced cell growth inhibition and prevented mitochondrial superoxide generation (Figures 1B and 1C).

**Figure 1:**
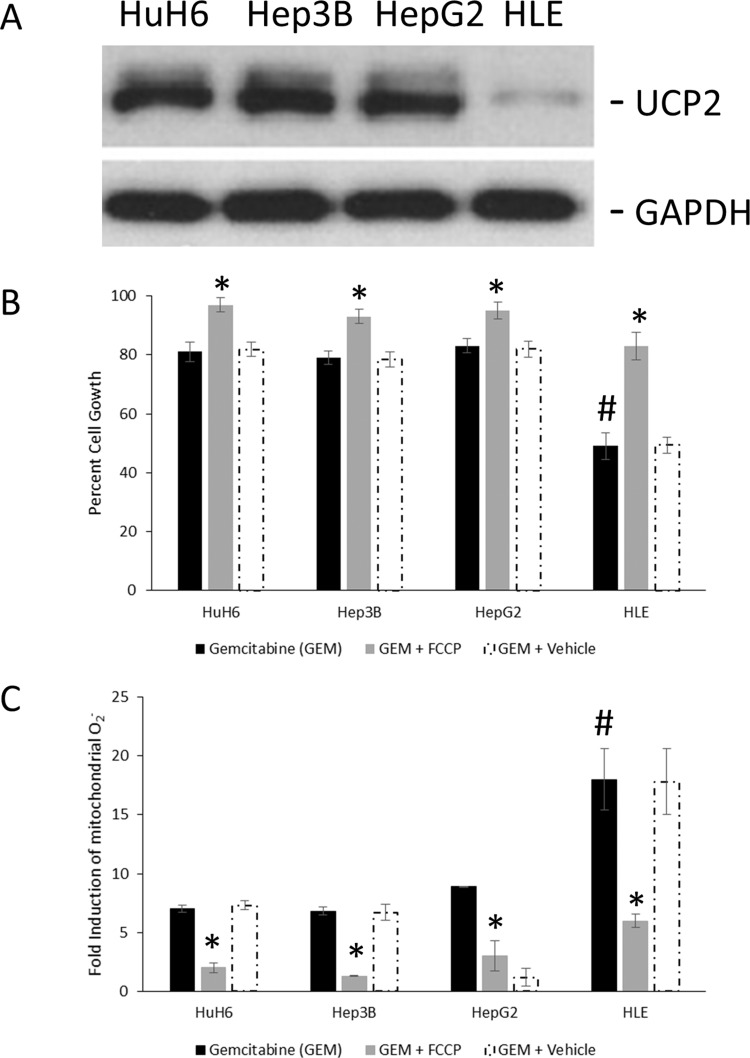
UCP2 expression levels in liver cancer cell lines dictate sensitivity to GEM-induced inhibition of cell growth (**A**) Basal expression levels of UCP2 in mitochondrial extracts obtained from indicated HCC cell lines. The blot was stripped and probed with GAPDH to serve as a loading control. (**B** and **C**) Effects of GEM on cell growth (**B**) and mitochondrial superoxide production (**C**) in the absence or presence of FCCP or vehicle (mock). Cells seeded in 96-well plates were treated with 1 μM GEM for 48 h alone or in the presence of 1 μM FCCP or 95% ethanol for 48 h (**B**) or 16 h (**C**). Statistical analysis: *P<0.05 GEM or GEM + vehicles compared with GEM + FCCP; #*P*<0.05 GEM in HLE compared with HepG2, Hep3B or HuH6 cells.

### Effect of modulating *UCP2* levels on sensitivity to gemcitabine treatment

Since HLE cells showed low endogenous UCP2 expression and HuH6 cells exhibited robust endogenous UCP2 protein expression, these two cell lines were chosen for the subsequent gain- and loss-of-function studies respectively. Successful overexpression in HLE cells and siRNA-mediated silencing using two different siRNAs targeting *UCP2* in HuH6 cells were confirmed by immunoblotting (Figure 2A). Ectopic overexpression of *UCP2* reversed sensitivity to GEM-induced cell growth inhibition (Figure 2B) and significantly decreased mitochondrial superoxide production (Figure 2C) in HLE cells. Conversely, siRNA-mediated silencing of *UCP2* with two different siRNAs targeting *UCP2*, but nor a scrambled control siRNA, significantly increased sensitivity to GEM-induced cell growth inhibition and mitochondrial superoxide generation (Figures 2B and 2C). Since HCC cell lines have highly heterogeneous UCP2 expression levels (Figure 1A), we next determined whether therapeutic targeting of *UCP2* is a feasible option only in those instances when it is in abundance or inhibition of even smaller amounts as found in HLE cells has an impact on cell growth. As shown in Figure 2(D), siRNA-mediated knockdown of even the basal level of UCP2 in HLE cells increased sensitivity to GEM-induced cell growth inhibition. This was accompanied by an increase in mitochondrial superoxide production (result not shown). Cumulatively, our data indicated a central role of UCP2 expression on potential success of using GEM to inhibit cell growth in HCC cell lines.

**Figure 2:**
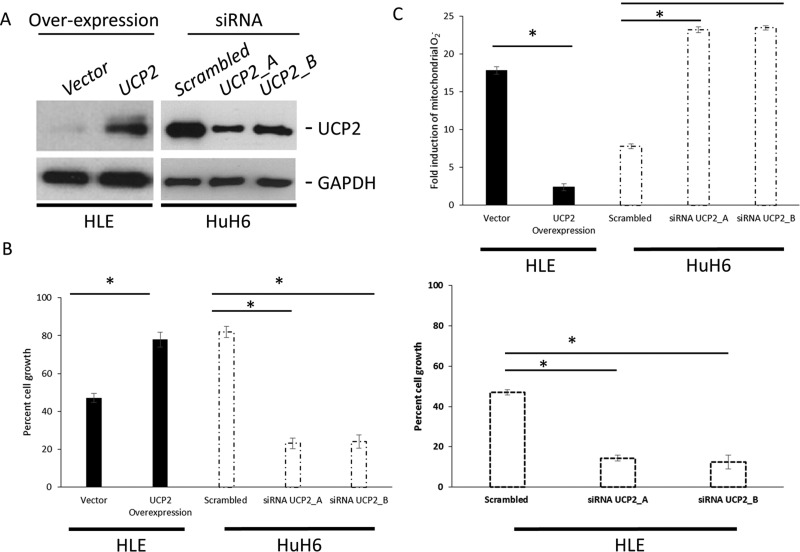
Modulating *UCP2* expression level changes sensitivity to GEM (**A**) Expression levels of UCP2 in HLE and HuH6 cells 60 h after transfection with overexpression vector or with two different siRNAs targeting *UCP2* respectively. (**B** and **C**) Effects of modulating *UCP2* expression on GEM-induced inhibition of cell growth (**B**) and mitochondrial superoxide production (**C**). Cells seeded in 96-well plates were transiently transfected 12 h before being treated with 1 μM GEM for 48 h (**B**) or 16 h (**C**). (**D**) Effects of *UCP2* silencing on GEM-induced inhibition of cell growth in HLE cells. Cells seeded in 96-well plates were transiently transfected 12 h before being treated with 1 μM GEM for 48 hs. Statistical analysis: *P<0.05 Vector compared with overexpression or siRNA scrambled compared with *UCP2* siRNA A and B.

### Knockdown of *UCP2* sensitized HuH6, Hep3B and HepG2 cells to apoptosis

It has been reported that modification of *UCP2* could sensitize certain cancer cell for chemotherapy in leukaemia cells [21]. Moreover, *UCP2* also inhibits cell apoptosis induced by ROS. This led us to examine whether disruption of *UCP2* in HuH6, Hep3B and HepG2 cell could sensitize the cells to apoptosis induction. Apoptosis induction was evaluated by determining caspase-3 and caspase-7 activity. Caspase-3 and caspase-7 activities were higher in each of the three cell lines post transfection of either of two siRNAs targeting *UCP2*, but not the scrambled control (Figure 3). Taken together, it demonstrated that knockdown of *UCP2* expression sensitized the cells to apoptosis.

**Figure 3:**
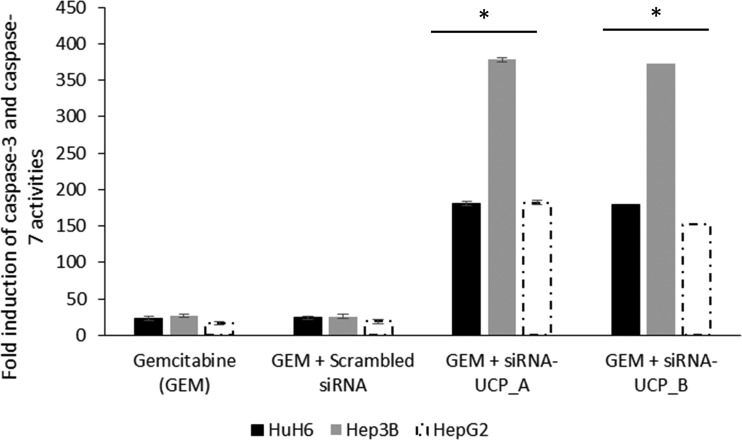
Silencing the expression of *UCP2* in HuH6, Hep3B and HepG2 cells induced apoptosis post-GEM treatment as assessed by downstream caspase activity Cells, seeded in 96-well plates, were transiently transfected 12 h before being treated with 1 μM GEM. The FLICA fluorescence intensity, corresponding to the level of caspase-3 and -7 activities, was measured. Values are the means (± S.D.) of three independent experiments each performed in triplicate. Statistical analysis: *P<0.05 *UCP2* siRNA A or B compared with scrambled siRNA.

## DISCUSSION

UCP2 is known to suppress ROS level which is overexpressed by various types of cancer cells including HCC cell lines. However, the role it plays in HCC cell lines as well as its relation with drug-resistance is not well defined yet. In the present study, relative expression level of endogenous UCP2 dictated sensitivity to GEM-induced inhibition of cell growth. In addition, inhibition of the *UCP2* expression sensitized HCC cell lines with robust UCP2 protein expression to apoptosis induction, which implied that *UCP2* may be a therapeutic target for chemotherapy in hepatic carcinoma (Figure 4).

**Figure 4:**
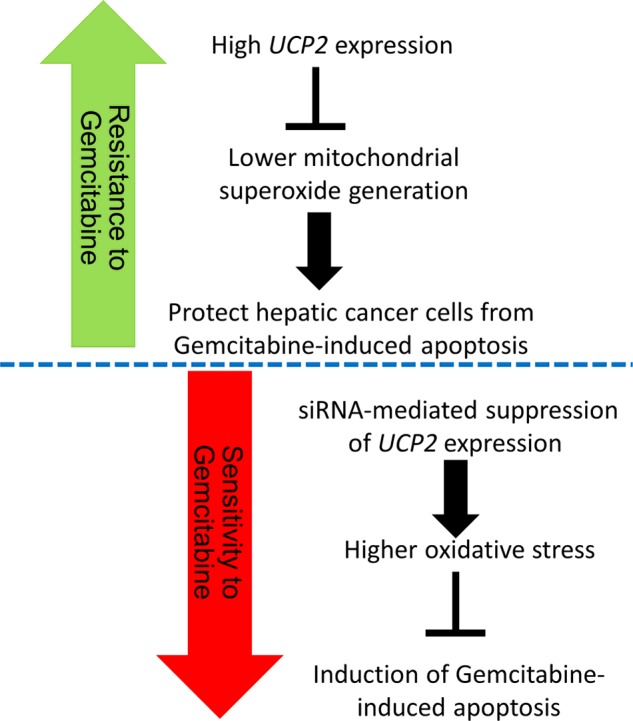
Model illustrating the relationship between expression level of *UCP2*, mitochondrial superoxide generation and sensitivity to GEM-induced cell growth inhibition in HCC cell lines

In the current study, we have used siRNAs to silence UCP2 expression. An alternative to this would be to use chemical inhibitors. There are two known classes of chemical inhibitors of UCP2, one the more commonly tested extract from *Gardenia jasminoides* known as Genipin [22] and the other developed chromane derivatives [23]. Even though Genipin has been shown to be a highly selective inhibitor of UCP2, numerous studies have elucidated that Genipin specifically impedes UCP2-mediated proton leak in brain tissue, kidney mitochondria and pancreatic β-cells [24,25]. To the best of our knowledge, the chromane derivatives are still not commercially available. Genipin, even though it might be a highly cost effective and therapeutically viable alternative to using siRNAs, will need to be tested in future studies to determine if they have effect on hepatic tissues as well.

It had been demonstrated that cancer cells employed numerous strategies to nullify toxicity generated by chemotherapeutic drugs [26]. Among these strategies, turning over the ROS levels by induction of *UCP2* represents a major adaptive response in cancer cell [26]. Several studies had confirmed that inhibition of *UCP2* in cancer cell sensitized drug-resistant cancer cells to cytotoxic agents [21,22]. Those data also suggested these drugs sensitize cancer cell mainly by increasing ROS level which could further result in cell death. Our data corroborates the same.

On the other hand, in addition to *UCP2*’s role in the cellular ROS level, previous studies also demonstrated *UCP2* could regulate the function of p53 [16,27], the pivotal tumour suppressor which cause apoptotic cell death in response to cellular stress stimuli [16,28]. Unlike some HCC cell lines which express the p53 mutant (Huh7) or have p53 deletion (Hep3B) [29], studies had confirmed that HepG2 cell processes a functional wild type p53 and activation of p53 in HepG2 cell could be used as novel approach to identify the genotoxic damage [30,31]. Since p53 and nuclear factor-κ-gene binding (NF-κB)-dependent apoptosis had been demonstrated in HepG2 cell [32], it is possible that inhibition of *UCP2* in HepG2 cell could restore the p53 function therefore to sensitize the cell for apoptosis induction. It however remains to be determined what causes sensitization in the Hep3B and HuH6 cell lines. Whether *UCP2* could act as a p53 antagonist in HepG2 cell and how *UCP2* inhibit p53 function merits further investigation. Moreover, although our study indicated that targeting *UCP2* alone may be an innovative strategy for cancer therapy, earlier studies have shown that the chemosensitivity was varying among different HCC cell lines [26]. Hence, it is possible that inhibition of *UCP2* could act synergistically with other chemotherapeutic agents for HCC therapy. In conclusion, our study provides new insight for the role of *UCP2* in HCC.

## Abbreviations

FLICA: fluorescent labeled inhibitor of caspase
GAPDH: glyceraldehyde-3-phosphate dehydrogenase
GEM: gemcitabine
HCC: hepatocellular carcinoma
ROS: reactive oxygen species
UCP2: uncoupling protein 2
